# Dermoscopy of skin infestations and infections (entomodermoscopy) – Part I: dermatozoonoses and bacterial infections^☆^^☆☆^

**DOI:** 10.1016/j.abd.2021.04.007

**Published:** 2021-10-05

**Authors:** Renato Marchiori Bakos, Clarissa Reinehr, Gabriela Fortes Escobar, Leandro Linhares Leite

**Affiliations:** a Universidade Federal do Rio Grande do Sul, Porto Alegre, RS, Brazil; b Postgraduation in Medical Sciences, Universidade Federal do Rio Grande do Sul, Porto Alegre, RS, Brazil; c Hospital de Clínicas de Porto Alegre, Porto Alegre, RS, Brazil; d Service of Dermatology, Hospital São Lucas da Pontifícia Universidade Católica do Rio Grande do Sul, Porto Alegre, RS, Brazil

**Keywords:** Bacterial infections, Dermoscopy, Skin diseases

## Abstract

Dermoscopy is an essential *in vivo* diagnostic technique in the clinical evaluation of skin tumors. Currently, the same can also be said about its implications when approaching different clinical situations in Dermatology. A growing number of reports on dermatological scenarios and diseases have been published, in which dermoscopy has been of great diagnostic help. The term “entomodermoscopy” was coined to describe dermoscopic findings in skin infestations and also in dermatoses of infectious etiology. In part I of this article, the main dermoscopic descriptions of zoodermatoses and bacterial infections will be addressed. In many of them, such as scabies, pediculosis, myiasis, and tungiasis, it is possible to identify the pathogen and, consequently, attain the diagnosis more quickly and use the technique to follow-up therapeutic effectiveness. In other situations that will be described, dermoscopy can allow the observation of clinical findings with greater detail, rule out differential diagnoses, and increase the level of confidence in a clinical suspicion.

## Introduction

Dermoscopy is a noninvasive method used in the investigation of an increasing number of skin symptoms and dermatological clinical scenarios. The popularity of this method is mainly due to its practical use, good accuracy and also because it is accessible to most physicians who evaluate the skin. It allows the *in vivo* observation of a new morphological world and, thus, the identification of structures that are not visible to the naked eye, which contributes greatly to the detection of these dermatoses. It was called in the past “the dermatologist's stethoscope” because of its assistance in daily clinical practice.^1^

Originally described for the assessment of pigmented lesions and for the differential diagnosis of skin tumors, the use of dermoscopy has been expanded and in recent decades there have been many reports or series of cases employing it in the study of dermatoses of infectious-parasitic origin, as in this review.^2^ The identification of dermoscopy structures requires a learning period, similarly to when it is applied in the evaluation of pigmented lesions. It is noteworthy that the clinical variability shown by some infectious-parasitic diseases makes some dermoscopic patterns and structures reported in these diseases have varying degrees of reproducibility by other observers, depending on the clinical scenarios and the patients’ specific situations. To describe the most accepted dermoscopy findings, the International Society of Dermoscopy has recently carried out a consensus on dermoscopy used in infectious and inflammatory diseases.^3^

Dermoscopic findings should always be associated with the patients’ symptoms and clinical morphological characteristics. It is essential for the best performance of dermoscopy in clinical practice to combine it with a good anamnesis and dermatological physical examination. The present article aims to demonstrate the main uses of dermoscopy in the identification of infectious-parasitic diseases.^2^ In part I of this study, the dermoscopic patterns of dermatozoonoses and bacterial infections will be described.

## Dermatozoonoses

### Scabies

Scabies is a contagious dermatosis caused by the mite *Sarcoptes scabiei* var. hominis. It occurs worldwide and it has no predilection for gender, age or ethnic group. After mating, the male mite dies and the female enters the epidermis. It moves around creating the scabies tunnels, in which it eliminates eggs and waste (feces). Itching in these areas can be intense, especially at night. Clinically, the presence of erythematous papules, sometimes linear or serpiginous (scabies tunnel), is frequently observed, often covered by hematic crusts resulting from excoriations.^4^ The most frequently affected locations are interdigital spaces, wrists, axillary pillars, inframammary, periumbilical and genital regions. Nodular lesions can occur, as well as eczematization or secondary impetiginization. Although common, certain cases of scabies can be initially treated as another type of dermatosis, delaying its diagnosis.

**Figure 1:**
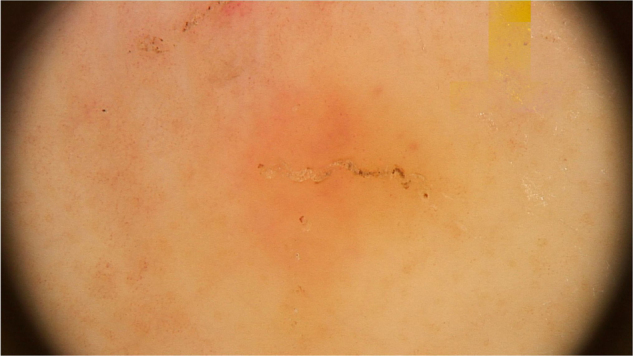
Dermoscopic image of scabies showing a brownish triangular structure followed by a whitish linear structure forming the “jet with contrail” image. (FotoFinder, original magnitude ×20) Source: Authors' personal collection.

The dermoscopic findings of scabies were first described by Argenziano et al. In their study, they identified a brown triangular structure (in the shape of a “hang glider”) in the anterior part of a whitish serpiginous area that was called “jet with contrail” (Fig. 1) in 93% of 70 patients. The brownish structure corresponding to the anterior portion of the mite and the whitish structure to the scabies tunnel. This description is a milestone in entomodermoscopy, as it is the first report on the practical applicability of dermoscopy for the diagnosis of infectious-parasitic diseases.^5^ These data were reproduced in other studies. Dupuy et al. compared dermoscopy with the traditional method of skin scarification and observation of the fragments under an optical microscope after preparation. Dermoscopy showed high sensitivity (91%) and specificity (86%) and faster performance, in addition to being the technique preferred by the patient.^6^ Currently, it can be considered one of the main screening methods for scabies, as it allows the identification of the causative agent, which is the main criterion to establish the diagnosis according to the 2020 International Alliance for the Control of Scabies (IACS) Consensus Criteria for the Diagnosis of Scabies.^7^

**Figure 2:**
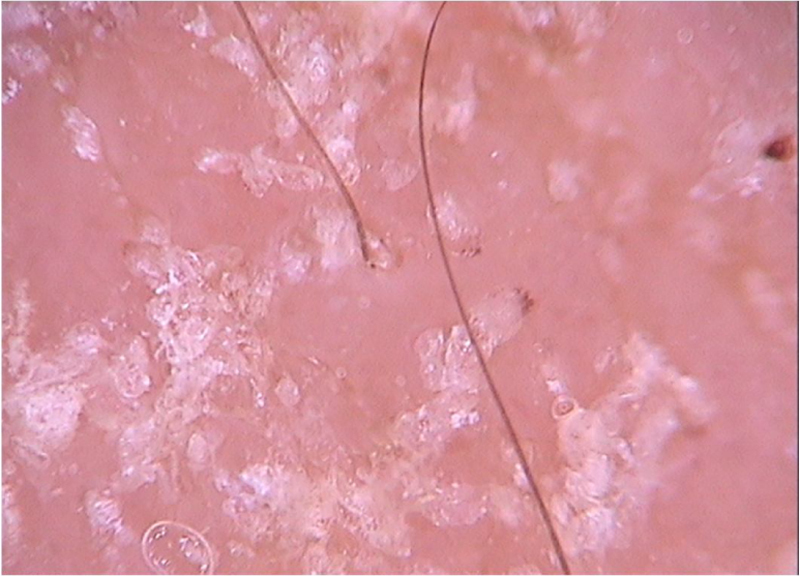
Dermoscopic image of erythroderma caused by scabies demonstrating the “noodle sign”. (FotoFinder, original magnitude ×20) Source: Hospital de Clinicas de Porto Alegre collection.

In peculiar clinical situations, such as immunosuppressed patients, individuals with chronic neurological conditions, among others, the mites can disseminate and constitute the crusted scabies variant, also called Norwegian scabies. Clinically, erythematous scaling plaques can be observed and even erythroderma. Dermoscopy can identify an enormous amount of tunnel-shaped structures with a brownish triangular structure. They can sometimes crisscross, establishing a noodle pattern (Fig. 2).^8^ Many patients can have pruritus or prurigo nodularis lesions for weeks after treatment. Dermoscopy can also be of great help in the therapeutic follow-up, allowing the observatio of viable mites or ruling out their presence after treatment.

### Myiasis

Myiasis is a consequence of infestation by fly larvae and can be classically classified as furuncular or cavitary. Infestation by *Dermatobia hominis* fly larvae on the skin usually characterizes furuncular myiasis, a frequent form of disease manifestation, especially in rural areas. It is a common dermatosis throughout tropical America. The fly eggs reach the skin transported by phoretic insects, such as mosquitoes, and penetrate through the hair follicles or through the bite perforation. The cycle continues with the development of the larvae in the subcutaneous tissue (it can last up to 30 days) until it reaches a stage ready to emerge and become pupae in the soil.

**Figure 3:**
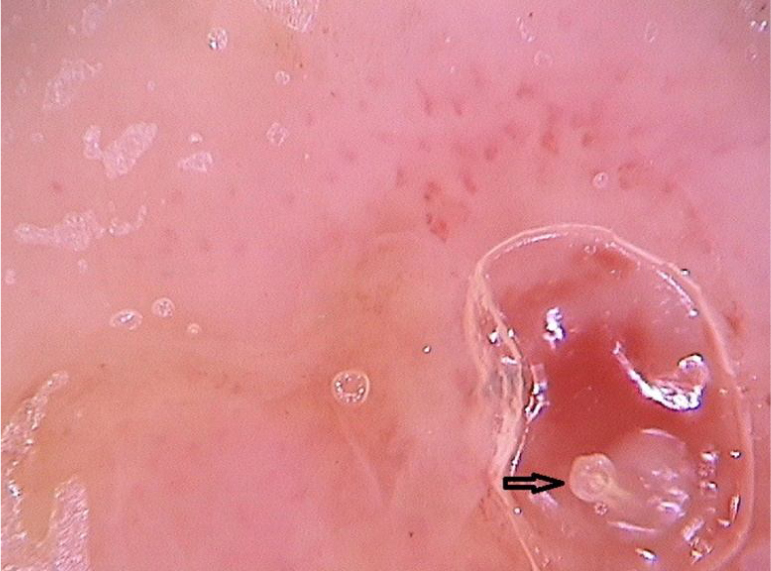
Dermoscopic image of furuncular myiasis demonstrating the presence of the anterior portion of the *Dermatobia hominis* larva. (FotoFinder, original magnitude ×20) Source: Hospital de Clinicas de Porto Alegre collection.

Clinically, it manifests as an erythematous, painful nodule with a central orifice that eliminates a serous secretion, often mistaken as abscessed lesions. Secondary infections may occur and, therefore, their suspicion and detection are important. Dermoscopy can be helpful in this process, as it allows larva identification.^9^ Its anterior portion, where the respiratory system (spiracle) is located, is visible *in vivo* through the cavity orifice. It recurrently shows movements towards the surface to breathe. Contact dermoscopy causes occlusion of the orifice and, consequently, the larva starts moving to the surface more frequently, thus being identified. It is possible to observe a yellowish central structure with spikes around it, depending on the size of the larva. It is less frequently found in the early stages of its development (Fig. 3). There have also been reports of larva observation with non-contact dermoscopy using polarized light equipment; however, it is possible that the observation time must be longer to identify the larva movements. These findings also help in the treatment of furuncular myiasis, since occlusion for a few hours leads to larva death. Occlusion of the orifice with adhesive tape or similar makes it difficult for the larva to breathe, and dermoscopy helps in the observation of the absence of respiratory movements.^4^, ^9^

Cavitary myiasis usually occurs in wounds, necrotic areas, or tumors. Poor hygiene or individuals with special social conditions such as advanced age or people with psychiatric or neurodegenerative diseases may also be more predisposed to it. Clinically, it is characterized by the presence of a large number of larvae in the same cavity. In Brazil, the most often associated species is the *Cochliomyia hominivorax* fly. On dermoscopy, the larvae are immediately identified and can be seen as yellowish in color, with a spiracle at one end and rings of blackish spicules.^10^

### Pediculosis

Pediculosis is characterized by an infestation of the scalp by the head lice *Pediculus humanis capitis*. It occurs all over the world, sometimes as outbreaks in schools or places where there is great crowding of people. The detection of head lice is crucial for the diagnosis, however, due to the speed at which they move, they cannot always be seen. Thus, it is common for diagnostic suspicion to rely on the occurrence of symptoms such as itching on the scalp and posterior cervical region. Moreover, clinically it is possible to observe erythematous papules with hematic crusts, sometimes excoriated, in these areas, as well as the observation of nits adhered to the hair shafts. Secondary impetiginization and eczematization are frequent.

**Figure 4:**
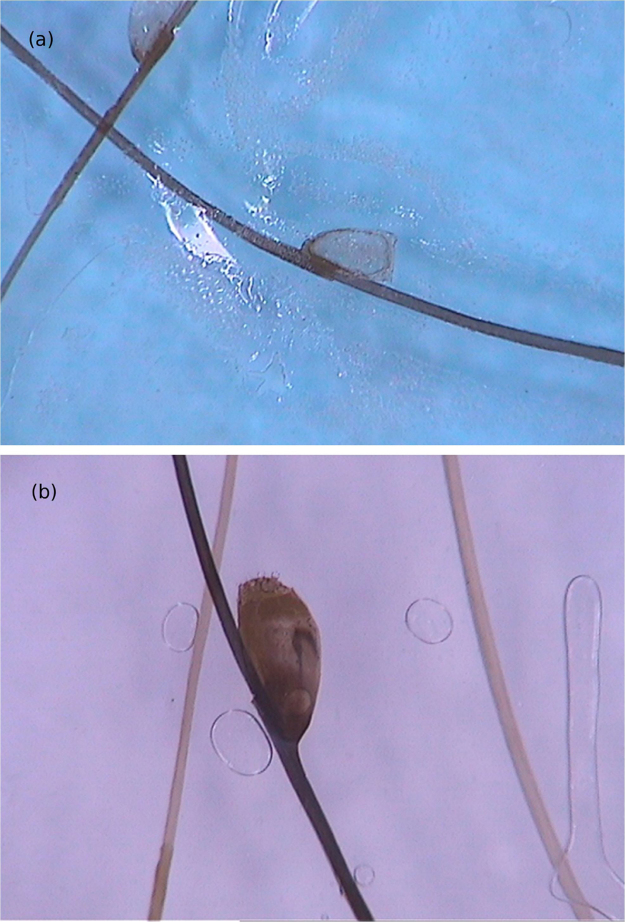
Dermoscopic image of pediculosis, showing empty (A) and viable (B) nits. (FotoFinder, original magnitude ×20) Source: Hospital de Clinicas de Porto Alegre collection.

Di Stefani et al. described for the first time the use of dermoscopy to help in the diagnosis of pediculosis. Using a polarized light dermatoscope, they demonstrated that it was possible to differentiate *in vivo* the nits from keratin debris adhered to hair shafts derived from scaly scalp eruptions, such as seborrheic dermatitis or psoriasis. In addition, it was possible to observe that some nits were empty with a fracture in one of their extremities, while others were still intact and filled with viable lice.^11^ Another report pointed out the possibility of using *ex vivo* dermoscopy to identify nits. Hair shafts with whitish structures appear cut off and it is also possible to identify scaling and viable or empty nits (Fig. 4).^12^

Pubic pediculosis or phthiriasis is caused by the *Phthirus pubis* louse. It is more frequently observed in young adults, usually acquired through sexual contact. Fomites such as sheets and towels can also transmit the causal agent. Pruritus in the genital area is the most relevant symptom and, depending on the degree of the infestation, it can spread to other body areas. Excoriated erythematous papules are frequent. Dermoscopy allows easy identification of lice. They can be found with their claws attached to hairs, often with the anterior portion invading the skin. Similar to pediculosis of the scalp, nits are attached to hairs and can be found to be empty or still viable.

### Tungiasis

Tungiasis is a self-limited ectoparasitosis, endemic to tropical and subtropical areas, originated from South and Central America, with subsequent dissemination to Africa and Asia. It is caused by the female *Tunga penetrans* flea, which inhabits dry and sandy soils, and penetrates the skin of its hosts (humans, dogs, cats, and pigs) after direct contact with the soil. It has a high prevalence in low-income areas of endemic countries, with a lack of paved streets and the habit of walking barefoot. It is often more common in children, particularly males.^13^

The flea enters the epidermis and feeds on blood from the dermal vascular plexus.^14^ It is considered to be the smallest of all flea species (with a maximum length of 1 mm), and gravid females can reach up to 1 cm inside the host, where it remains for a period of up to 5 weeks. This exponential growth occurs within the first 2 weeks, followed by the laying of eggs by the flea and its death.^15^

As sand fleas are considered poor jumpers (they can jump up to 20 cm), the area most commonly affected in hosts is the feet, preferably the subungual edge, although any site on the body can be affected.^13^, ^15^ It usually presents as a yellowish-white papule, with a dark brown center. It is often accompanied by symptoms such as pruritus, pain, and foreign body sensation.^15^ Secondary bacterial infection is a common complication and, occasionally, tetanus can be a serious complication.^13^

*Tunga penetrans* is a macroscopic insect and many of its structures are visible to the naked eye. Dermoscopic findings usually correspond to anatomical elements of the flea, such as the external structures of the exoskeleton or even internal visceral structures.

From a dermoscopic point of view, a pigmented annular structure with a central pore is usually present in most lesions. Its color can vary from light brown to almost black and corresponds to the pigmented chitin that surrounds the opening at the posterior end of the insect's abdomen.

The host's blood inside the flea intestines can be seen on dermoscopic examination. The bluish-gray spots and the reddish convoluted tubules are the dermoscopic findings corresponding to hematin. Videodermoscopic demonstration of the flea intestinal peristalsis *in vivo* moving such structures corroborates the findings. These structures are not found in all cases and may not be seen on the examination.

**Figure 5:**
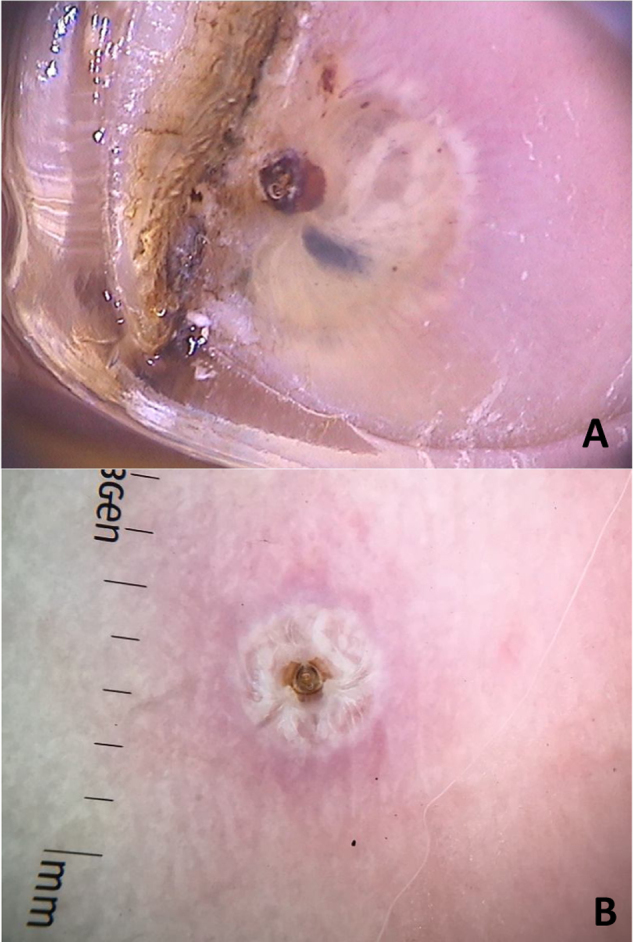
Dermoscopic images of tungiasis demonstrating the chitin ring with a bluish-gray spot (hematin) around it and whitish structures in a chain distribution (A) and the central chitin ring surrounded by whitish structures (eggs) (B). (FotoFinder, original magnitude ×20) Source: Hospital de Clinicas de Porto Alegre collection and authors' personal collection.

Structures described as “white chains” are frequent dermoscopic findings and correspond to eggs within the gravid female abdomen.^16^ Eggs are visible as small whitish oval structures, usually organized in a chain inside the flea, forming this characteristic finding. In addition, it is not unusual for eggs that have already been eliminated by the female to be seen in the area outside the lesion (Fig. 5).^16^

More recently, another finding has been described as silver dendritic fibers, characterized by silver-colored fibrous structures connected to the central pigmented ring. These structures would probably correspond to the flea trachea, which would connect to spiracles located in the central pigmented pore. Moreover, dermoscopic analysis allows showing the *T. penetrans* activities *in vivo*, such as breathing through the spiracles and defecation. Visceral peristalsis has also been recorded on videos in the literature.^17^

In endemic areas, tungiasis is easily recognized and treated with simple excision, often by the patients themselves or by the primary care physician.^13^ However, the lesions have a differential diagnosis with viral warts, myiasis, furuncles, abscesses, paronychia, arthropod bites, folliculitis, foreign body granulomas, and fungal granulomas, which can constitute a diagnostic challenge, especially in non-endemic areas, where clinicians are unfamiliar with the disease or in cases with atypical presentations. Dermoscopy is a non-invasive, fast and low-cost method that helps in the identification, aiming to avoid complications and the continuation of its life cycle.

### Larva migrans

Cutaneous larva migrans is a parasitic disease caused by hookworm larvae after contact with soil contaminated by cat or dog feces.^14^ The main agent is the *Ancylostoma braziliense*, but several species are involved, such as *Ascaris suum* and *Bunostomum phlebotomum*. These larvae are unable to penetrate the basement membrane of human skin and remain confined to the epidermis, causing lesions as the larvae travels through the skin. Clinically, they present as serpiginous, erythematous, pruritic lesions usually in the lower extremity where contact with soil is more frequent.^14^, ^18^

There are reports of observation of larva and its structures with dermoscopy.^14^, ^18^ Although the ×40 magnification was enough to detect larva, the ×10 magnification was not helpful for this diagnosis.^19^

**Figure 6:**
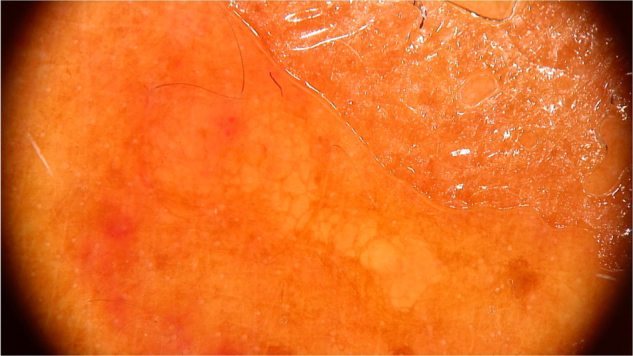
Dermoscopic image of *larva migrans* showing translucent, structureless areas in a segmental arrangement. (FotoFinder, original magnitude ×20) Source: Authors' personal collection.

Dermoscopy disclosed translucent, structureless, brown areas with a yellowish periphery.^14^, ^18^ They present a segmental or oval arrangement.^14^ These findings corresponded to the body of the larva and were confirmed by near-infrared fluorescence imaging observations. The empty path resulting from the passage of the larva was noticed as red dotted vessels (Fig. 6).^14^

Although anamnesis and physical examination are sufficient for diagnosis, the dermoscopic findings can be useful in unclear cases.

### Disseminated strongyloidiasis

Disseminated strongyloidiasis is a helminthic infection caused by *Strongyloides stercoralis*, which occurs more frequently in immunocompromised patients and has high mortality rates. Although cutaneous manifestations are uncommon, when present they may be relevant for diagnostic suspicion and dermoscopy is useful in these cases.^20^ The incidence of disseminated strongyloidiasis is increasing, with most cases being associated with the use of systemic corticosteroids or other immunosuppressive agents. Systemic corticosteroids are associated with *Strongyloides stercoralis* hyperinfection, increasing the risk of developing the disseminated form of the disease by threefold, even at low doses and for short periods. Additionally, patients with impaired cell immunity, such as patients with hematologic malignancies and those infected with the acquired immunodeficiency virus (HIV) or human T-cell lymphotropic virus (HTLV-1), are at increased risk of developing disseminated strongyloidiasis. *Strongyloides stercoralis* infection starts with penetration of the filariform larva through the skin in contact with contaminated soil. In immunosuppressed patients, the infection cycle is accelerated, leading to a high parasite load and symptomatic disseminated infection. In these cases, several organs are affected, including the skin.

**Figure 7:**
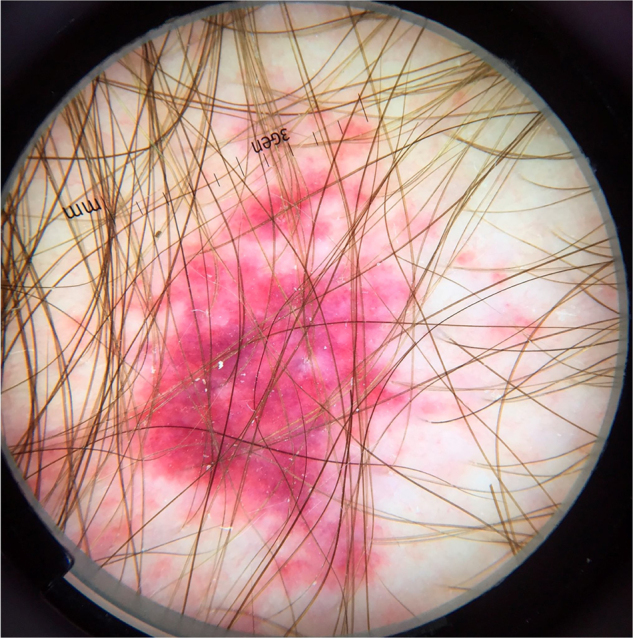
Dermoscopic image of disseminated strongyloidiasis showing homogeneous purpuric areas. (FotoFinder, original magnitude ×20) Source: Hospital de Clinicas de Porto Alegre collection.

The most common cutaneous symptom of *Strongyloides stercoralis* infection is larva currens, a linear, urticarial pruritic lesion at the site of the active penetration of the larva into the skin. In the disseminated form, the cutaneous manifestations result from the hematogenous spread of the parasite: purpura and periumbilical and thigh petechiae, resulting in the so-called “fingerprint” pattern.^21^ Periumbilical purpura occurs due to involvement of the dermis by *Strongyloides stercoralis* larvae that migrate from the vessel wall to the dermal connective tissue, due to the extravasation of erythrocytes, and the periumbilical location is a result of retrograde venous flow. Dermoscopy of these purpuric lesions highlights the presence of homogeneous purpuric areas, corresponding to hemorrhagic areas (Fig. 7).^22^, ^23^ These findings are based on few case reports; however, dermoscopic findings may be useful to rule out other dermatological diseases. The definitive diagnosis of disseminated strongyloidiasis involves the identification of the larva, whether in the anatomopathological study of the skin, in bronchoalveolar or gastric lavage, or in tracheal secretions, depending on the patient's symptoms. Histopathological analysis of purpuric skin lesions shows a mild lymphocytic infiltrate and the presence of larvae in the dermis.

### Demodecidosis

Demodecidosis is an infection of the human pilosebaceous unit associated with Demodex, an ectoparasitic mite that affects the face and scalp.^24^ There are two species involved in human infection: *Demodex folliculorum* and *Demodex brevis*, and it is estimated that the prevalence of Demodex infection in humans varies from 24% to 100% in healthy individuals. Infestation should always be considered in cases of perioral dermatitis or in patients with treatment-refractory rosaceous lesions. Lesion presentation may also resemble seborrheic dermatitis and pustular folliculitis.^24^

Clinically, the infection manifests as roughness of the skin (spinulosis), erythema, papules, and/or pustules, usually associated with pruritus and a burning sensation. The primary form occurs when there is no pre-existing inflammatory skin disease and the secondary form occurs when there is an abnormal increase in the amount of Demodex mites in patients with a pre-existing dermatosis in the area, or even due to long-term use of topical corticosteroids. The most important differential diagnoses are rosacea and seborrheic dermatitis, and folliculitis, perioral dermatitis, and acne should also be included.^24^, ^25^ The definitive diagnosis of demodecidosis is made by the microscopic identification of the parasite, which takes time and requires specific equipment and a qualified professional.^25^

**Figure 8:**
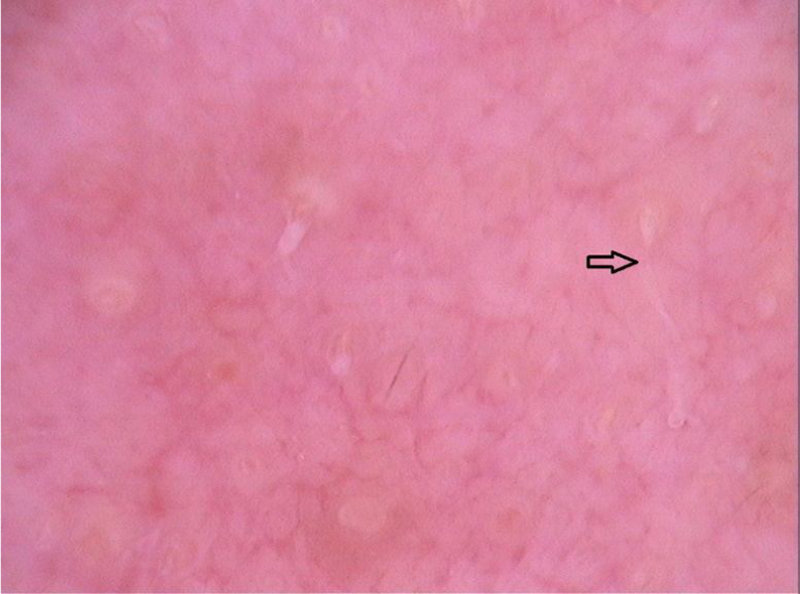
Dermoscopic image of demodicidosis showing “Demodex tails”. (FotoFinder, original magnitude ×20) Source: Authors' personal collection.

Dermoscopy can help in the differential diagnoses. As an example, the pattern seen in seborrheic dermatitis, which includes dotted vessels and fine yellowish scaling, is not commonly found in demodecidosis.^24^ Segal et al. conducted a study with 72 patients who had erythematous eruptions on the face, of which 55 had demodecidosis. On dermoscopy, 54 patients had “Demodex tails” and follicular openings containing Demodex. Microscopic confirmation of the diagnosis of demodecidosis was possible in 52 patients, and agreement between the findings of the two methods was quite high. “Demodex tails” are non-follicular and perifollicular gelatinous filaments that protrude from the follicular openings and represent the presence of the mite (Fig. 8). Follicular openings with Demodex are dilated follicular openings containing plugs of gray-brown amorphous material surrounded by an erythematous halo. Both findings are specific for the diagnosis of demodecidosis.^24^, ^25^

In patients with inflammatory variants of demodecidosis, reticulated horizontal dilated vessels have also been observed, but this finding is non-specific for this dermatosis.^25^ In pustular forms, the findings are also non specific, with a purulent collection measuring 1 to 10 mm in diameter, surrounded by an erythematous halo.^25^ Dermoscopy can also be used to monitor response to treatment, as the aforementioned structures may be absent or less frequently present on dermoscopic analysis.^25^

## Bacterial infections

### Leprosy

Leprosy is a chronic infectious disease caused by *Mycobacterium leprae*, which is endemic in Brazil and other developing countries.^26^ It has a broad clinical spectrum, varying from the paucibacillary tuberculoid pole to the multibacillary lepromatous pole. This variation is based on the patient's immune response and makes it difficult to correctly identify the disease.^26^ Moreover, atypical presentations and leprosy reactions can occur, further delaying the diagnosis.^26^, ^27^, ^28^

Although the suspicion of leprosy is assessed by a set of signs and symptoms detected on physical examination and data from the anamnesis, dermoscopy was recently incorporated as a tool that can be useful in its identification in certain cases. Therefore, several findings have been described, varying according to the clinical forms.

**Figure 9:**
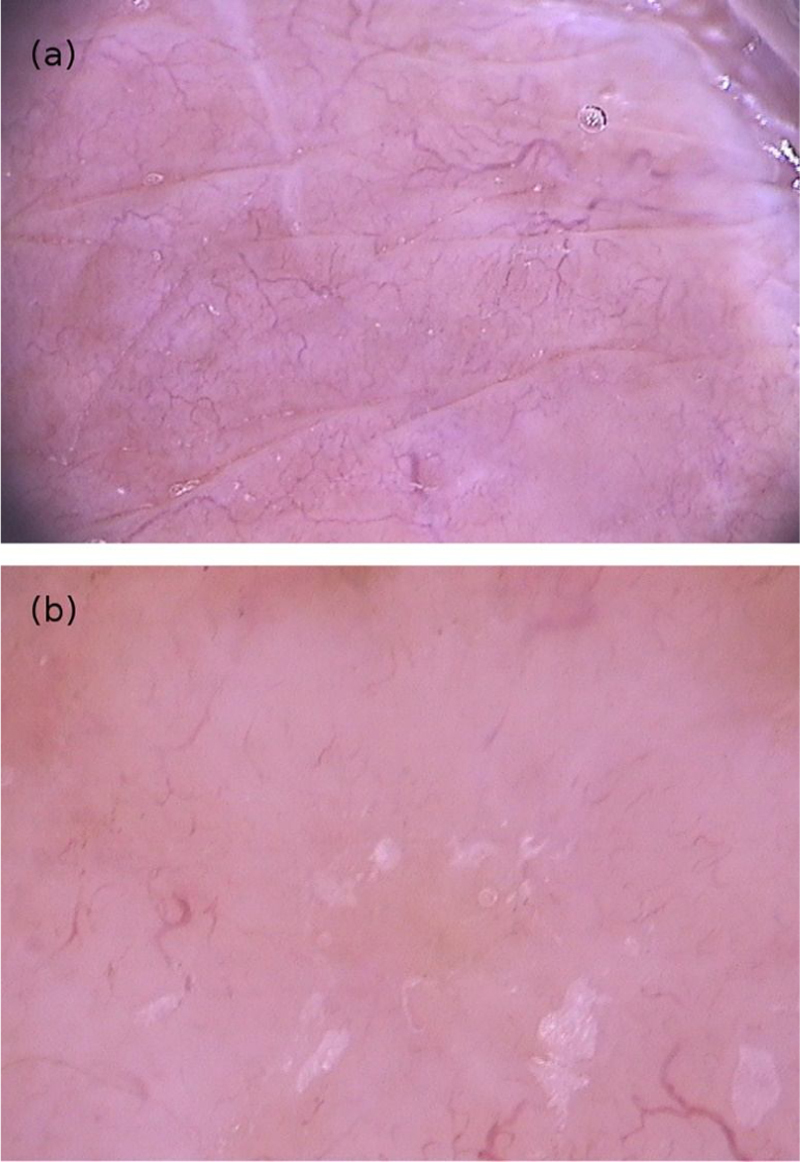
(A), Dermoscopic image of borderline tuberculoid leprosy showing yellow-orange areas surrounded by erythema and telangiectasias with linear branching; and (B), dermoscopic image of the histoid variant multibacillary leprosy showing a papular lesion with diffuse yellowish color and multiple heteromorphic telangiectasias. (B) (FotoFinder, original magnitude ×20) Source: Hospital de Clinicas de Porto Alegre collection.

Tuberculoid leprosy (TL) is a clinical form limited by the host’s good immune response, exhibiting few lesions that clinically present as well-defined annular plaques with raised edges and a hypochromic center.^26^, ^27^ Dermoscopy of the edge of the lesions showed structureless, yellow-orange areas surrounded by linear branching erythema and telangiectasias associated with a decreased pigmented network, loss of hair follicles, and reduction of white dots (sweat gland ostia) (Fig. 9A).^27^ These dermoscopic findings were similar to those found in the borderline tuberculoid (BT) form.^27^, ^28^, ^29^ Additionally, white areas, interpreted as a disease-induced reduction in melanocytes, have been frequently seen in BT lesions.^28^, ^29^ Histopathologically, these cases have shown a scarcity of cutaneous adnexa, dermal granulomas and vascular ectasias. The latter two would correspond, respectively, to the yellow-orange areas and telangiectasias observed on the dermoscopy.^27^, ^29^

Bordeline lepromatous leprosy (BLL) and lepromatous leprosy (LL) show on dermoscopy typical yellowish-orange areas as in other granulomatous dermatoses and reduction of cutaneous adnexa, with areas of atrichia.^27^ Despite this similarity regarding the dermoscopic findings in these cases, it likely is the diffuse histiocytic infiltrate that explains its occurrence. BLL also showed a reduced or absent pigment network, which has been reported as linear areas similar to chrysalides, whose observation can help differentiate it from other granulomatous dermatoses.^27^ On the other hand, an increase in the pigment network has been described in LL, which is associated with dilated branched vessels. Moreover, scaling associated with cutaneous xerosis has also been observed.^27^, ^29^

LL can present with nodular lesions called hansenomas, which present on dermoscopy with a diffuse yellowish coloration, a discrete brownish halo, and, in the center of the lesion, a pearlescent cicatricial aspect surrounded by multiple heteromorphic telangiectasias.^26^, ^30^ This dermoscopic pattern is similar to that found in histoid leprosy lesions,^27^, ^31^, ^32^, ^33^ a rare form of lepromatous leprosy characterized by high bacillary load and by nodules and plaques on apparently normal skin (Fig. 9B).^33^, ^34^ However, the histoid form lesions, in turn, showed bright white structures similar to those seen in dermatofibromas, melanomas, and basal cell and squamous cell carcinomas.^31^, ^32^ These structures would only be seen under polarized light dermoscopy and may correspond to fibrotic alterations of the dermis.^31^ On histopathology, hansenomas showed numerous macrophages and vascular ectasia, which would explain, respectively, the nodular appearance and telangiectasias on dermoscopy.^30^

Loss of body hair including the eyebrows is a common event in leprosy, with approximately three-quarters of multibacillary patients presenting with madarosis. Several trichoscopic findings compatible with non-cicatricial alopecia have been described in the eyebrows, the main ones being reduced capillary density, multiple vellus hairs, and distortion of skin pigmentation. Next, white pinhead dots surrounded by target pigmentation and structureless areas of yellowish-white color were the most common findings.^35^

Additionally, hyperpigmentation induced by clofazimine, a drug included in the polychemotherapy regimens for multibacillary patients, has been described on dermoscopy as a characteristic honeycomb pattern, with yellow and white globules interspersed over a darkened background.^29^

Leprosy reactions have also been studied using dermoscopy. These conditions may occur before, during, or even after regimen completion.^36^ Also called reverse reaction, the type 1 leprosy reaction often affects dimorphic clinical forms and is the consequence of a cellular immune response associated with late hypersensitivity.^26^ On dermoscopy, the lesions have a diffuse erythematous background,^27^, ^29^, ^36^ associated with yellowish-orange^27^, ^36^ or reddish-orange areas.^36^ Arboriform vessels, short linear fine vessels, and blurred linear vessels have also been observed.^29^, ^36^ The presence of white scaling and follicular plugs has also been reported.^27^, ^36^ Blurred vessels seem to be a specific finding and it has been postulated they are caused by an increase in the number of lymphocytes and loss of the normal organization of the granuloma.^36^

Type 2 leprosy reaction, also called erythema nodosum leprosum, is a result of immune complex deposition and is typical of multibacillary patients.^26^ It courses with lesions on the extensor surfaces of the limbs, sometimes associated with systemic symptoms.^26^ Dermoscopic findings were non specific and corresponded to increased erythema and branched telangiectatic blood vessels.^27^, ^29^

As with other non-infectious granulomatous diseases, the presence of structures described as orange or yellow in color on dermoscopy in cases of leprosy is strongly associated with the presence of dermal granulomas and their consequent mass effect.^29^ Telangiectatic vessels and their presentation pattern usually vary according to the etiology. In granuloma annulare, the vessels show a dotted pattern, and in lipoid necrobiosis they are arboriform; in lupus vulgaris and sarcoidosis they show linear ramifications. We noted that this variation is repeated within the clinical spectrum of leprosy, with vascular patterns varying according to the clinical form. In addition to these two main alterations, which are common to other granulomatous skin diseases, pigmentary disorders and loss of skin appendages are additional, more specific findings that indicate the diagnosis of leprosy.

The prevalence of leprosy has been steadily decreasing worldwide since the advent of polychemotherapy.^26^ However, this millenary disease is still endemic in several regions of Brazil and the world. As demonstrated, dermoscopy can contribute to its *in vivo* diagnosis, a crucial step to interrupt the chain of contagion.

### Syphilis

Syphilis is an infectious disease, transmitted mainly through sexual contact but also vertically, which is caused by *Treponema pallidum*, and has had a significant resurgence in the last two decades. Secondary syphilis occurs after the pathogen has disseminated through the blood or lymphatic route, usually 6 to 8 weeks after resolution of the primary lesions. It is characterized by a symmetrical, macular or papular, usually scaling eruption on the trunk, extremities, palms, and soles.

The variety of typical and atypical presentations of secondary syphilis makes this disease known as “the great imitator” and makes its diagnosis difficult, with a wide range of differential diagnoses. Of these, the main ones include pityriasis rosea of Gilbert, actinic porokeratosis, erythema annulare centrifugum, granuloma annulare, subacute lupus, discoid lupus, and certain annular variants of psoriasis.^37^ Dermoscopic study of the lesions can help in their identification and differentiation from these other dermatoses. Up to the present moment, only case reports have been published on the topic.

In cases of scaling skin lesions, the identification of Biett's sign or collarette can be facilitated by dermoscopy and has been described, particularly in lesions of palmar location, as a whitish circular scaling area with outward progression.^37^, ^38^ Although it is visible to the naked eye, dermoscopy allows its detection in smaller lesions, where the collarette sign is not clinically evident.^39^ They characteristically present with a diffuse orange or yellowish-red background.^37^, ^38^ It has been postulated that it might correspond to the extravasation of red blood cells and hemosiderin deposits that occur in secondary syphilis lesions.^38^ Its presence could help differentiate it from conditions that can confound and delay the diagnosis of syphilis, such as palmar psoriasis in its papular presentation, porokeratosis, and lichen planus.^38^ Moreover, a case of scalp lesions has been described showing circular scaling with inward progression on dermoscopy.^40^

Palmoplantar involvement is one of the most common presentations of secondary syphilis, and some of these diseases may involve this region, making differentiation difficult.^37^, ^38^ Additionally, dotted vessels, which have been found in some cases of secondary syphilis, are characteristic findings of psoriasis. Therefore, the identification through clinical inspection and/or dermoscopy of Biett's collarette and an orange background are more specific findings of secondary syphilis in these locations.^41^

Syphilitic alopecia (SA) can also be a manifestation of secondary syphilis. It is classified as non-cicatricial alopecia that may be associated with other cutaneous-mucous syphilis symptoms (symptomatic SA) or be the sole clinical element of the infection (essential SA).^42^, ^43^ Although SA most commonly affects the scalp, the hair from any area of the body may be affected.^42^ Trichoscopy has shown to be an important diagnostic tool in patients with alopecia of unknown origin and can help identify SA, especially when skin lesions typical of syphilis are not present. The absence of ‘exclamation mark’ hairs can help exclude alopecia areata, a condition that often resembles SA.^43^ There are four clinical patterns of SA: (1) The “moth-eaten” pattern: the most common and characteristic; (2) Diffuse pattern: similar to an acute telogen effluvium; (3) Mixed pattern: a combination of the two abovementioned patterns; (4) Alopecia of the eyebrows.^42^

Areas of alopecia in the “moth-eaten” pattern seem to exhibit specific features on trichoscopy: a reduced number of terminal hairs, with thin hair shafts similar to emerging vellus hairs invisible to the naked eye (<20 µm diameter), or even atrichia has been demonstrated.^42^, ^43^ Several empty follicular ostia are evident, even in the occipital region of the scalp, which can help differentiate it from androgenetic alopecia.^42^, ^43^

Other non-infection-specific findings have been reported as black dots, hypopigmentation of hair shafts, yellow dots, irregularly dilated blood capillaries, broken and “zigzag” hair shafts.^42^, ^43^, ^44^, ^45^ A reddish-brown pigmentation in areas of scalp alopecia may be present but it is not evident in areas of alopecia of the beard and eyebrows.^42^, ^45^ This pigmentation has regressed after antibiotic therapy and it has been speculated that it was due to an immune response-induced small vessel vasculitis against *T. pallidum* located in the blood vessels of the perifollicular epithelium.^42^

Finally, syphilis is a highly prevalent disease, with serious implications in several organs and systems. Despite its potential severity, syphilis is usually quite effectively treated with penicillin. Disease identification and administration of adequate treatment can prevent the severe systemic complications of syphilis. The dermoscopic and trichoscopic findings described above improve the early detection of diagnostic “clues”, allowing greater clinical suspicion and prompt interruption of the natural course of the disease through the implementation of therapeutic measures.

### Folliculitis

**Figure 10:**
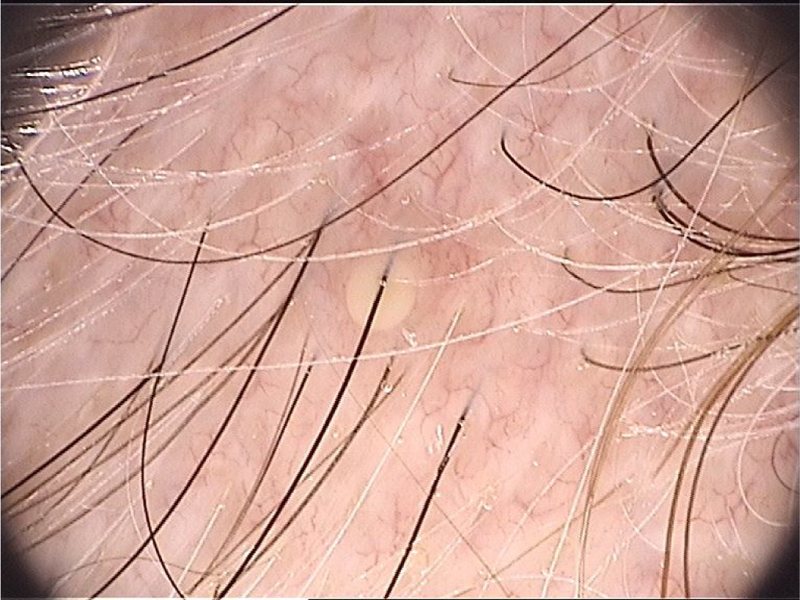
Dermoscopic image of a follicular pustule surrounded by bacterial folliculitis erythema. (FotoFinder, original magnitude ×20) Source: Authors' personal collection.

Folliculitis is a group of inflammatory diseases that can have an infectious origin or not. It occurs in hairy areas and, clinically, it discloses a papular-pustular eruption over an erythematous base, often accompanied by pruritus. Treatment varies depending on the identification of the causative agent. A prospective series evaluated the dermoscopic findings of 240 cases of folliculitis. The most affected areas were the face (35% of cases), trunk (30.4%), and scalp (21.3%). The vast majority of cases were of infectious origin (90%), with a diagnostic accuracy by dermoscopy of 72%.^46^ Depending on the etiological agent, dermoscopic findings might vary. In the case of folliculitis of bacterial origin, usually caused by *Staphylococcus aureus*, the main finding consisted of centrofollicular pustules, surrounded by erythema or dotted vessels (Fig. 10). Others, such as folliculitis caused by pityrosporum, scabies or demodecidosis showed the usual structures already described in this paper.

A case report has also identified the presence of centrofollicular pustules in a case of folliculitis caused by *Pseudomonas aeruginosa*.^47^ In these cases, it is important to add the clinical history of contact with environments potentially contaminated by the pathogen, such as hot tubs, swimming pools, or inflatable pool objects or sponges. Moreover, a papulopustular eruption in intertriginous areas or those covered by swimwear is also a typical presentation.

### Trichobacteriosis

Trichobacteriosis, formerly called trichomycosis, is a superficial bacterial infection caused by bacteria of the genus *Corynebacterium sp*.^48^ It can occur alone or in association with other corynebacterioses, such as erythrasma and keratolysis punctata. Previously attributed to *C. tenuis*, its main agent is *C. flavescens*.^48^ Others that have also been described include *C. propinquum* and *Dermabacter hominis*.

Clinically, it presents with yellowish concretions firmly adhering to the hair shafts, particularly in moist regions of the body, such as the axillary and pubic areas.^48^ They may rarely be reddish or blackish in color. It is often associated with an unpleasant odor and elimination of pigment in sweat that often stains clothes. Its main predisposing factors are hyperhidrosis, obesity, and poor hygiene.^48^

On dermoscopy, yellowish or translucent waxy or soft concretions are described, adhering along the entire length of the hair shafts.^49^, ^50^ Forms showing interruptions between the concretions may also be found.^50^ Concretions have been described as having a nodular, flame, brush, or feather shape.^49^

Although the diagnosis is most often clinical, its presentation can be confused with other conditions that course with structures adhered to the hair shaft. Because they clinically simulate lice nits, these structures were called pseudonits and correspond to conditions that make the differential diagnosis with pediculosis, such as trichobacteriosis itself, white piedra, black piedra, and the casts present in inflammatory conditions, such as psoriasis, seborrheic dermatitis, cutaneous lupus erythematosus, and lichen planopilaris. Trichoscopy allows an easy differentiation between these conditions and would quickly allow the diagnosis of trichobacteriosis when there are clinical doubts.^49^ Wood's lamp (yellow fluorescence), direct examination in Potassium Hydroxide (KOH), and Gram staining, in addition to confirmation by culture, are additional methods; however, they may not be necessary.^48^

Treatment consists of removing hair from the affected areas and optimizing hygiene. Topical antibiotics, such as erythromycin or clindamycin, may also be used.

## Final considerations

Dermoscopy can add valuable information when evaluating a range of zoodermatoses or bacterial skin infections. The dermoscopic findings described herein are based on the identification of a series of etiological agents of these diseases, which makes their diagnosis unequivocal. In others, dermoscopy helps to identify the clinical implications of the pathogen’s presence on the skin. In these cases, clinical correlation is crucial for diagnostic clarification, whether based on anamnesis data or peculiarities in the physical examination. If, on the one hand, there are dermoscopic patterns closely associated with some of the described dermatoses, it can be observed that, to date, the descriptions are based on isolated reports or series of cases for other nosological entities, which would be insufficient to determine a uniform pattern for all. Therefore, it becomes an interesting area to be further explored scientifically. It is worth mentioning that many of the lesions caused by infectious agents have areas of skin discontinuity and are potentially contaminated. To prevent the dermatoscope from becoming a fomite that will transmit the infection, it is recommended to use devices polarized light devices, without contact with the skin. When using devices with polarized or non-polarized light that make contact with the skin, cleaning the device with antiseptics must be carried out immediately after its use. The use of an insulating material adhered to the dermatoscope lens is another possibility, although there is some loss in the quality of the imaging of the examined area.

## Financial support

None declared.

## Authors' contributions

Renato Marchiori Bakos: Article design; article organization; drafting of the manuscript; review and approval of the final version of the manuscript.

Clarissa Reinehr: Drafting and editing of the manuscript; review and approval of the final version of the manuscript.

Gabriela Fortes Escobar: Drafting and editing of the manuscript; review and approval of the final version of the manuscript.

Leandro Linhares Leite: Drafting and editing of the manuscript; review and approval of the final version of the manuscript.

## Conflicts of interest

None declared.
